# Implementation of a Renal Precision Medicine Program: Clinician Attitudes and Acceptance

**DOI:** 10.3390/life10040032

**Published:** 2020-03-26

**Authors:** Katherine M. Spiech, Purnima R. Tripathy, Alex M. Woodcock, Nehal A. Sheth, Kimberly S. Collins, Karthik Kannegolla, Arjun D. Sinha, Asif A. Sharfuddin, Victoria M. Pratt, Myda Khalid, David S. Hains, Sharon M. Moe, Todd C. Skaar, Ranjani N. Moorthi, Michael T. Eadon

**Affiliations:** 1 Department of Medicine, Indiana University School of Medicine, Indianapolis, IN 46202, USA; kspiech@iu.edu (K.M.S.); purnimaranitripathy@gmail.com (P.R.T.); alwoodco@indiana.edu (A.M.W.); nesheth@iu.edu (N.A.S.); ksburges@iu.edu (K.S.C.); kkannegolla1@IUHealth.org (K.K.); adsinha@iu.edu (A.D.S.); asharfud@iu.edu (A.A.S.); smoe@iu.edu (S.M.M.); tskaar@iu.edu (T.C.S.); rmoorthi@iu.edu (R.N.M.); 2 Department of Medical and Molecular Genetics, Indiana University School of Medicine, Indianapolis, IN 46202, USA; vpratt@iu.edu; 3 Department of Pediatrics, Indiana University School of Medicine, Indianapolis, IN 46202, USA; khalidm@iu.edu (M.K.); dhains@iu.edu (D.S.H.)

**Keywords:** chronic kidney disease (CKD), hypertension (HTN), genetic testing, pharmacogenomics, APOL1

## Abstract

A precision health initiative was implemented across a multi-hospital health system, wherein a panel of genetic variants was tested and utilized in the clinical care of chronic kidney disease (CKD) patients. Pharmacogenomic predictors of antihypertensive response and genomic predictors of CKD were provided to clinicians caring for nephrology patients. To assess clinician knowledge, attitudes, and willingness to act on genetic testing results, a Likert-scale survey was sent to and self-administered by these nephrology providers (N = 76). Most respondents agreed that utilizing pharmacogenomic-guided antihypertensive prescribing is valuable (4.0 ± 0.7 on a scale of 1 to 5, where 5 indicates strong agreement). However, the respondents also expressed reluctance to use genetic testing for CKD risk stratification due to a perceived lack of supporting evidence (3.2 ± 0.9). Exploratory sub-group analyses associated this reluctance with negative responses to both knowledge and attitude discipline questions, thus suggesting reduced exposure to and comfort with genetic information. Given the evolving nature of genomic implementation in clinical care, further education is warranted to help overcome these perception barriers.

## 1. Introduction

Chronic kidney disease (CKD) and essential hypertension (HTN) affect roughly 15% and 30% of the general American population respectively [1,2]. While CKD is often concomitant with the occurrence of HTN, not all individuals with HTN develop CKD. The gap in knowledge regarding the causes of CKD and its association with other medical conditions is now being filled by emerging data on genetic determinants (which may be predictive of disease progression or therapeutic drug response) [3,4]. The perceived knowledge of, attitudes toward, and willingness to act on this genetic information are unknown among clinicians caring for nephrology patients. This study seeks to assess provider’s survey responses prior to the implementation of a broad genetic testing protocol.

Single nucleotide polymorphisms (SNPs) are the most common type of genetic variation and occur when there is a mutation at a single base pair. The etiologies of many diseases have been attributed to SNPs, and their identification has contributed to the pathophysiology of different types of CKD [3,5]. For example, the presence of susceptibility variants in particular gene loci (e.g., the loci of *UMOD* and *APOL1)*, may increase the risk of CKD and increase the possibility of end stage renal disease (ESRD) in specific populations [6,7]. Namely, *APOL1* polymorphisms, which are found as homozygous recessive risk alleles in approximately 10% of the African American population, are now recognized as risk factors that increase the progression of hypertensive and glomerular kidney diseases [7]. Additionally, other genetic predictors such as variations in *UMOD*, *SHROOM3*, *PDILT*, and others, have been uncovered in the CKDgen consortium [8]. Without the identification of these variants, many cases of CKD would still be classified as secondary to hypertension or an unknown etiology.

SNPs also contribute to an individual’s pharmacogenomic profile, and thus influence overall drug effectiveness, therapeutic index, adverse drug reactions, and other kinetic processes [9,10,11]. The Clinical Pharmacogenetics Implementation Consortium (CPIC) has reported approximately 350 drug-gene pairs (110 of which are identified as actionable pairs (this indicates the SNP requires altered drug selection or dosing)); however antihypertensive drug-gene pairs have not been endorsed yet [11,12]. Similarly, the Dutch Pharmacogenomic Working Group (DPWG) has recommended only one actionable antihypertensive drug-gene pair (i.e., *CYP2D6* and metoprolol) [13]. A lack of substantial support for antihypertensive pharmacogenomics has resulted in slower clinical implementation efforts that lag behind those of other drug-gene pairs [11].

In an effort to improve drug efficacy and reduce adverse events, clinical genetic and pharmacogenomic testing utilization has been promoted by physicians and researchers in a multi-hospital health system consisting of a university hospital and a county safety-net hospital. Within this health system, broad based panel testing was previously implemented for many CPIC level drug-gene pairs [14]. In 2018, implementation was expanded to test for variants in genes related to CKD progression, as well as pharmacogenetic variants predictive of antihypertensive agent efficacy [15]. Practitioners caring for patients with kidney disease received a panel of results on 72 variants in 26 genes relevant to CKD and hypertension pharmacogenomics. These results were uploaded to the health system’s electronic health record (EHR). While the providers may use this testing to change their practice, all changes in clinical care are at their behest.

In order to understand physician knowledge, attitude, and utilization of the genetic testing platform, physicians were surveyed and their responses were analyzed. We hypothesized that practitioners who cared for patients with renal disease would welcome the additional genetic data, which could enable the refinement of diagnosis and selection of the most efficacious antihypertensive agents. By performing a comparative analysis between survey question responses, an understanding of the physician’s knowledge, attitudes, and propensity to act on genetic data can be evaluated [16]. The ensuing analysis illuminated the barriers of the translation of clinical genetic testing and pharmacogenomics from research to everyday clinical practice.

## 2. Results

### 2.1. Implementation

Nephrologists practicing in a multi-hospital system, including a university and county safety-net hospital system, were approached for assent to genotype their patients for variants predictive of CKD and antihypertensive efficacy. The patients of assenting nephrologists were only genotyped after providing informed consent during their outpatient clinic visits. In most cases, results were provided to the clinician through the EHR within 2 weeks of the clinic visit. A complete list of genes, variants, and clinical phenotypes provided is included in Table 1. Evidence for these variants has been previously reviewed [11,17]. Variants with abundant evidence relevant to cardiovascular drugs were also included in the panel. The nephrologists and trainees completed baseline surveys prior to the genotyping of their first patient.

### 2.2. Demographics

A total of 76 respondents completed or partially completed the survey, of whom 51% (N = 39) were nephrologists and 49% (N = 37) were trainee residents or fellows who were rotating on a nephrology service. The survey response rate was 81%. Of the 76 respondents, 95% (N = 72) completed the entirety of the survey and 35% (N = 27) were female. The health care system of practice and the racial and ethnic breakdowns of the participants are given in Table 2. A total of 26 providers within the university health system and county safety-net hospital system received pharmacogenomic and genetic testing results for their patients. All surveys were completed prior to the implementation of genetic testing.

### 2.3. The Survey

Question category classification and the overall mean ± standard deviation agreement responses are illustrated in Table 3. Questions were stratified by discipline (i.e., Knowledge, Attitude, or Action) and one of three discussion topic categories. These categories included clinician opinions on genetics overall (General), genetic prediction of CKD progression (CKD), and genetic prediction of antihypertensive response (HTN). Responses were scored on a Likert scale from 1 to 5, with 5 indicating strong agreement.

Across the entire cohort of respondents, practitioners agreed that a patient’s genetic profile can influence CKD progression (Question 1 (Q1), 4.2 ± 0.6) and therapeutic response to antihypertensives (Q19, 4.0 ± 0.7). While the majority of respondents agreed that genetic makeup influences CKD progression and drug response, fewer respondents agreed there is sufficient evidence to implement genetic testing in patients with CKD (Q14, 3.2 ± 0.9). Despite this discrepancy, the majority of respondents agreed they would follow the dosing suggestions of a pharmacogenomic test for a new prescription (Q22, 4.0 ± 0.7). The questions with the lowest level of agreement were among those with the widest standard deviation of responses (Q11, Q16, and Q17). As such, practitioners were split as to whether genetic testing would increase patient anxiety, their comfort level in discussing results, and time limitations for holding such a discussion in clinic. Complete response comparisons are provided in Supplemental Table S1.

### 2.4. General Knowledge and Attitude Toward Genetics

**Figure 1:**
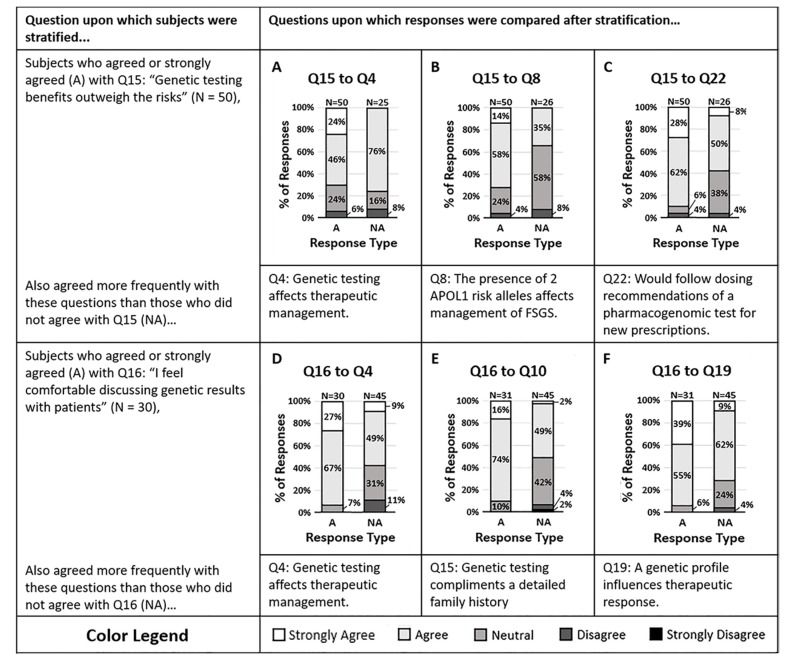
(**A**–**C**) Survey respondents who felt the benefits of genetic testing outweigh the risks (Q15) also agreed that (**A**) genetic testing of CKD patients provides information that may change therapeutic management (Q4, *p* = 0.013), (**B**) the presence of two APOL1 risk alleles in a patient would impact management of Focal Segmental Glomerulosclerosis (FSGS) (Q8, *p* = 0.0057), and (**C**) they would follow the dosing suggestions of a pharmacogenomic test for new prescriptions (Q22, *p* = 0.0018). (**D**–**F**) The majority of survey respondents who agreed they feel comfortable discussing genetic results with patients (Q16) also agreed that (**D**) genetic testing of CKD patients may affect therapeutic management (Q4, *p* = 0.0031), (**E**) genetic testing complements a detailed family history (Q10, *p* = 0.0010), and (**F**) a patient’s genetic profile can influence their therapeutic response to antihypertensives (Q19, *p* = 0.0038). Response colors are strongly agree (white), agree (light gray), neutral (medium gray), disagree (dark gray), and strongly disagree (black).

A sub-analysis of the main survey results sought to understand the impact of knowledge and attitude on a clinician’s willingness to act on genetic data. Clinicians were more likely to agree that genetic testing benefits outweigh the risks (Q15). Those who agreed that the benefits outweigh the risks were also more likely to agree that the presence of two *APOL1* risk alleles in one of their Focal Segmental Glomerulosclerosis (FSGS) patients would affect their therapeutic management (Q8, *p* = 0.006) and were more likely to agree that they would comply with the dosing suggestions of a pharmacogenomic test for new prescriptions (Q22, *p* = 0.002). Those who believe that the benefits outweigh the risks appear to be more willing to act on genetic data and comply with pharmacogenomic dosing suggestions. Of the individuals who agreed the benefits outweighed risks, the data suggested they are more willing to act on pharmacogenomic data than genetic testing data (Q4, *p* = 0.01, and Q8). Figure 1A–C illustrates the responses to other questions stratified by response to question 15.

Clinicians relayed a split response in attitude when asked if they felt comfortable discussing genetic results with their patients (Q16). Those who agreed and those who did not agree showed differences in their willingness to act on genetic data. The majority of clinicians who agreed that they felt comfortable discussing genetic results also agreed that genetic testing provides information that may influence them to change the therapeutic management of their CKD patients (Q4, *p* = 0.003). Clinicians who did not feel comfortable discussing results were proportionately less likely to agree that genetic testing may impact their therapeutic strategy (Q4). Figure 1D–F suggest that after stratifying clinicians by their comfort in discussing genetic results, the comfortable physicians were more likely to act on the results.

### 2.5. Genetics Predictors of Chronic Kidney Disease Progression

**Figure 2:**
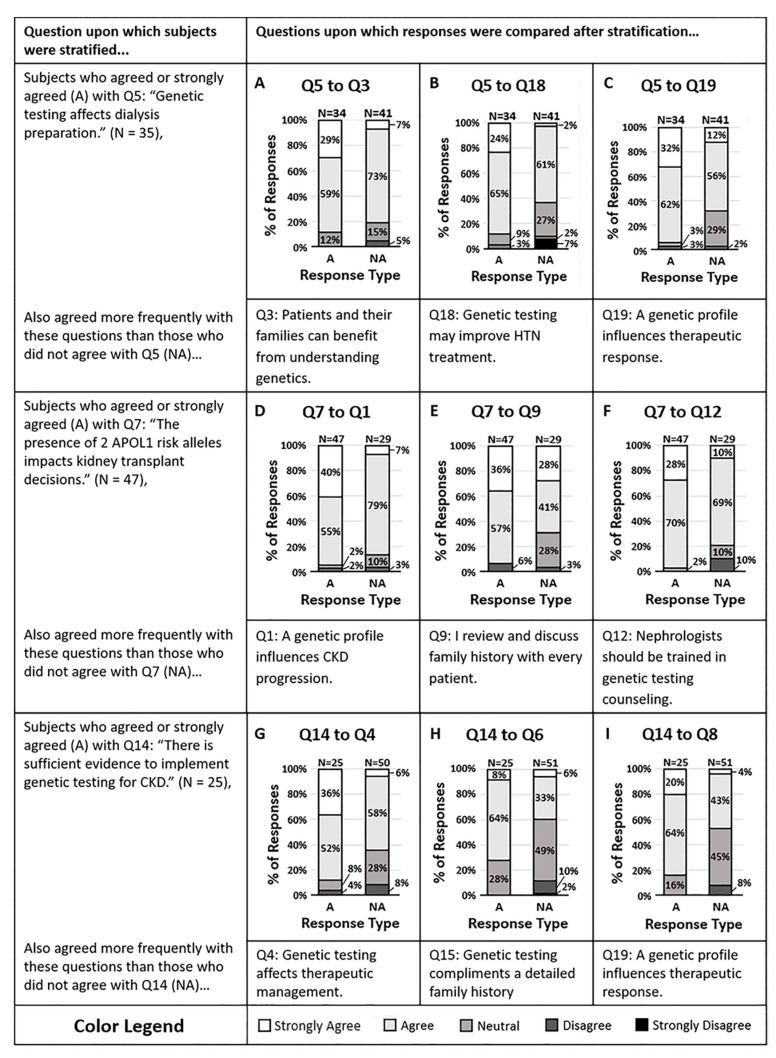
(**A**–**C**) Survey respondents who disagreed that genetic testing affects dialysis preparation (Q5) mostly agreed that (**A**) patients and their families can benefit from understanding the genetic contributors of CKD (Q3, *p* = 0.0473), (**B**) genetic testing may help them treat their patient’s hypertension more effectively (Q18, *p* = 0.0060), and (**C**) a genetic profile can influence antihypertensive therapeutic response (Q19, *p* = 0.0041). (**D**–**F**) Nearly all of the survey respondents who agreed that the presence of two APOL1 risk alleles in a potential donor impacts the decision for kidney transplant (Q7) also concurred that (**D**) a patients genetic profile can influence their risk of CKD progression (Q1, *p* = 0.0020), (**E**) they review and discuss family histories with every patient they meet in the hospital or clinic (Q9, *p* = 0.0011), and (**F**) nephrologists should be trained to interpret and counsel patients on genetic variants that contribute to CKD (Q12, *p* = 0.0149). (**G**–**I**) Survey respondents who disagreed that there is sufficient evidence to implement genetic testing in patients with CKD (Q14) were split approximately halfway on whether they agreed or disagreed that (**G**) genetic testing of CKD patients may affect therapeutic management (Q4, *p* = 0.0050), (**H**) genetic testing for CKD provides information that will help delay or halt CKD progression (Q6, *p* = 0.0138), and (**I**) the presence of two APOL1 risk alleles would impact FSGS management (Q8, *p* = 0.0052). Response colors are strongly agree (white), agree (light gray), neutral (medium gray), disagree (dark gray), and strongly disagree (black).

We examined whether a clinician’s propensity to act on genetic variants predicting CKD progression was associated with their attitude and knowledge of CKD risk variants (Figure 2). Respondents were split as to whether the genetic test may alter their dialysis preparation strategies (Q5). However, the majority agreed that the presence of two APOL1 risk alleles in a potential donor would impact the decision for kidney transplant (Q7). Of those who agreed and disagreed with question 5, the majority of respondents in both groups agreed that patients and their families would benefit from understanding their genetic risk factors of CKD (Q3, *p* = 0.05). Additionally, those who disagreed that *APOL1* status would impact organ transplant decisions (Q7) were also less likely to agree that genes influence CKD progression (Q1, *p* = 0.002). Despite having knowledge about the genetic risk factors of CKD and despite feeling that patients and their families would benefit from understanding these factors, some individuals were not willing to act on genetic data.

Using a knowledge question (Q14), we then stratified respondents into those who agreed and disagreed that there is sufficient evidence to implement genetic testing for CKD. Those who agreed that there is sufficient evidence were also more likely to agree that genetic testing provides information that may change the therapeutic management of their CKD patients (Q4, *p* = 0.005) and were more likely to agree that genetic testing will help them delay or halt the progression of CKD (Q6, *p* = 0.01). In contrast, those who disagreed with question 14 were split as to whether genetic testing might change their therapeutic management or delay or halt the progression of CKD in their patients. It appears that the more aware a physician is of evidence that supports genetic testing, the more likely they are to utilize genetic test results in their clinical practice.

### 2.6. Genetics Predicting Antihypertensive Response

**Figure 3:**
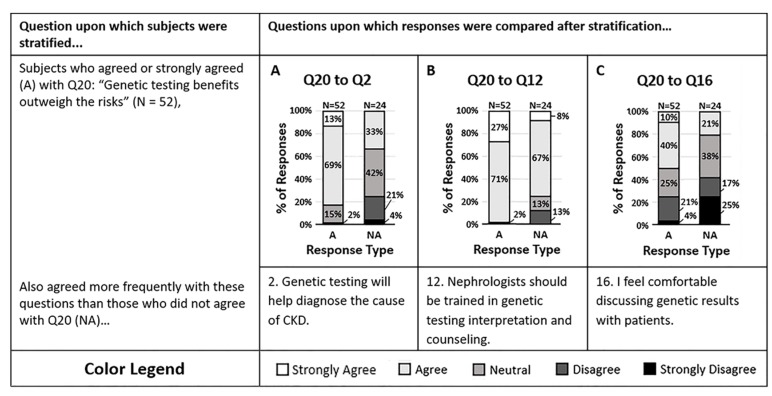
(**A**–**C**) Survey respondents who agreed that genetic testing of HTN patients provides information that may change which antihypertensives are prescribed (Q20) were more likely to agree that (**A**) genetic testing will help diagnose the cause of a patient’s CKD more effectively (Q2, *p* = 0.00004), and (**B**) nephrologists should be trained in genetic testing interpretation and patient counseling (Q12, *p* = 0.0040). However, those who agreed with question 20 were split halfway on whether they agreed or disagreed that (**C**) they feel comfortable discussing genetic test results with patients (Q16, *p* = 0.0194). Response colors are strongly agree (white), agree (light gray), neutral (medium gray), disagree (dark gray), and strongly disagree (black).

The final disease condition category of the survey assessed opinions on the use of pharmacogenomics to guide antihypertensive therapy (Figure 3). The majority of respondents agreed that pharmacogenomic testing provides information that may change the antihypertensives they prescribe (Q20). Those who agreed with question 20 were more likely to agree that genetic testing will help them diagnose the cause of their patient’s CKD more effectively (Q2, *p* = 0.00004) and were more likely to agree that nephrologists should be trained in CKD genetic testing interpretation and counseling (Q12, *p* = 0.004). However, those who agreed with question 20 were split in response when asked if they felt comfortable discussing genetic results with patients (Q16, *p* = 0.02). It appears that clinicians who agree with the potential value of genetic and pharmacogenomic tests are more likely to act on genetic data regardless of comfort.

Individuals who disagreed with question 20 were also more likely to disagree that genetic testing will help them determine the cause of their patient’s CKD more effectively. However, the majority still agreed that nephrologists should be trained in genetic testing interpretation and counseling. Despite personal therapeutic management opinions, it is suggested that the majority of all survey respondents saw value in a nephrologist’s ability to understand genetic testing data and ability to counsel patients on those results.

## 3. Materials and Methods

### 3.1. Participants

A genetic test was implemented in a multi-hospital system. All clinicians (practicing in nephrology clinics or nephrology service (N = 94) in the health system) who were approached had eligible patients and assented to the genotyping of their patients. In total, 76 respondents completed all or part of a survey about their knowledge, attitude, and willingness to act on genetic test results. The clinicians were classified as either nephrologists (i.e., nephrologists or academic attendings) or trainees (i.e., fellows and residents). Trainees were approached based on their participation in a nephrology service. The study was approved by the Institutional Review Board of Indiana University (IRB #1705413046).

### 3.2. The Survey

The survey consisted of 26 questions, 23 of which were opinion based. The remaining three questions assessed demographics of the respondents. Opinion based responses were graded on a LIKERT scale (i.e., “strongly disagree”, “disagree”, “neutral”, “agree” “strongly agree”). These responses were numbered 1, 2, 3, 4, and 5 respectively.

The survey was self-administered and confidential. The survey was sent via email or was hand delivered in a de-identified envelope to individuals who did not respond electronically [18]. No records of personal data (including email addresses) were saved, however records of completion or participation refusal were kept to ensure that individuals who refused were not contacted again. The responses were stored in RedCap, secured, and analyzed in Excel.

## 4. Analysis

Opinion based questions were divided into three clinical categories: general genetics, CKD risk genetics, or antihypertensive efficacy genetics. Questions were also divided by discipline: knowledge, attitude, and action. Of those 23 questions, six were identified as questions of primary interest. Namely, questions 5, 7, 14, 15, 16, and 20. These questions were selected based on response distribution and relational *p*-value significance.

The agree (i.e., “strongly agree” and “agree”) and did not agree (i.e., “strong disagree”, “disagree”, and “neutral”) responses to the questions of interest were compared to the agree and disagree responses of the remaining 22 questions in a contingency table [19,20].

## 5. Statistics

Each contingency table was analyzed via Fisher’s exact test in excel [16]. Additionally, all question responses were relayed as mean ± standard deviation of agreement.

To compare knowledge, attitude, and action responses, analyses that involved unlike discipline variables were prioritized for final interpretation (e.g., question 5 is an Action question, therefore analyses of interest would involve variable questions that are categorized as Attitude or Knowledge).

## 6. Discussion

Among the individuals queried on their opinions of precision medicine, many were willing to implement genetic testing in their clinical practice. The majority of respondents agreed that genetic profiles affect CKD progression and therapeutic drug response. However, the majority also agreed that there isn’t enough evidence to implement clinical genetic testing in patients who have CKD, despite suggesting that they might use a genetic test’s results to change the therapeutic management of their CKD patients. Additionally, there appears to be a debate on whether genetic testing can halt or delay CKD progression, which may be influenced by the fact that many believe current research has not produced enough affirmation that supports the benefits of genetic testing. Alternatively, or additionally, a perceived lack of therapeutic options that target the genetics of interest could drive the lack of enthusiasm [21].

Interestingly, the clinicians were in agreement that the benefits of genetic testing outweigh the risks, and that patients and their families can benefit from understanding the genetic factors of CKD. Individuals who believe that the benefits outweigh the risks are more willing to use clinical genetic and pharmacogenomic data. Nonetheless, it appears that more clinicians in our cohort favor pharmacogenomic testing over genetic testing for CKD risk stratification. When the clinicians were asked if they would follow the dosing suggestions of a pharmacogenomic test for new antihypertensive prescriptions, the majority agreed that they would comply.

One barrier contributing to the lack of support in clinical genetic testing is comfort. Many physicians, regardless of agreement stance towards CKD genetic tests, agreed that they were uncomfortable speaking with patients about genetic results. Generally, individuals who agreed that they were comfortable speaking with patients about genetic test results were more likely to agree that they would utilize the testing data. This may be because they feel confident in their knowledge about genetic testing, however more research is needed to support this postulation. Some individuals who are uncomfortable discussing genetic results may feel this way because they don’t see a net benefit in genetic testing when compared to current diagnostic and therapeutic practices. It has been discussed that cost and analytic accuracy in genetic tests are perceived barriers that many feel prohibit everyday clinical use, despite the potential benefits of improved patient treatment [22,23]. Observations, however, suggest that one way to overcome these barriers is through the education of providers on clinical genetics [24]. This notion is consistent with the data presented in this survey analysis: individuals who responded positively to having knowledge of clinical genetics and pharmacogenomics were more likely to act on the given genetic data.

Specifically, this study highlights that while the use of clinical genetic and pharmacogenomic tests are popular in other fields, genetic testing for CKD risk stratification has yet to gain broad traction among nephrologists. In addition, pharmacogenomic testing for antihypertensives, while not currently endorsed by CPIC or DPWG, is generally supported by clinicians throughout our health care systems. This is exemplified by the 26 providers who are currently utilizing the panel based genetic testing method mentioned previously.

Within the field of medicine, momentum is shifting from general reactive-based clinical practices to precision medicine, defined as personalized treatment based on an individual patient’s disease and needs [25,26,27]. Specialty practices outside of nephrology, such as oncology or psychiatry, have been faster to embrace advances in genome-wide sequencing, big-data analytics, and haplotype analysis [28,29,30]. Changes in therapeutic management behavior in peer practitioners is often a necessary step to normalize clinical genomic usage [31]. By observing the amount of willingness nephrologists had to implement genetic data in the survey, it is indicative that while precision medicine usage is not fully accepted yet, clinicians were more willing to use pharmacogenomics data, perhaps because of its perceived therapeutic benefit. Further studies are required to replicate genome-wide association studies and genotype-based pharmacokinetic studies [32]. For individuals who are still unsure of the net benefits of genetic testing, personalized pharmacogenomic analysis reports for physicians have shown changes in clinician perceptions towards utilizing pharmacogenomic services [24]. Personalized reports such as these are thought to positively influence clinician knowledge.

Our small sample size may limit the study’s ability to represent the general population of practicing clinicians. A second limitation is the correlative nature of the study (individuals who highly agree with one question were likely to agree with other similar survey questions). While the positive correlation and sub-group analyses have selection bias, these were only exploratory analyses. Our survey analysis also reflects the institutional biases and views of the authors. These limitations are counterbalanced by strengths. For example, by designing the survey to reflect some of the common principles of medical innovation (i.e., Knowledge, Attitude, and Action), a unique way to understand nephrologist’s current perception status of genetics and pharmacogenomics was introduced. This study also involved different health systems and locations. This provided a good mix of clinicians in academic and partly academic settings. Additionally, assessing trainee’s opinions allowed inclusion of those with contemporary medical training.

Future studies should aim toward randomized control trials for pharmacogenomic methods and *APOL1* testing. This will expand available evidence, and will allow a greater proportion of clinicians to formulate opinions on the value of genome-based prescribing and diagnosis in kidney care. In general, the nephrology providers queried were willing to use genetic data, particularly if it pertains to pharmacogenomics. However, significant barriers were still observed in educational resources and provider comfort; these limitations may be circumvented over time by increased evidence, education, and accessibility of/to pharmacogenomics and genetic testing for CKD stratification.

## Figures and Tables

**Table 1:** Phenotype—Genotype Interactions tested.

Gene	Variants Tested	Relevant Phenotype
***ADRB1***	rs1801252, rs1801253	Beta-Blocker Efficacy
***APOL1***	rs73885319, rs60910145, rs71785313	Risk of CKD
***CYP2C19***	rs4244285, rs4986893, rs28399504, rs72552267, rs41291556, rs6413438, rs12248560	Clopidogrel Efficacy
***CYP2C9***	rs1799853, rs1057910, rs28371686, rs9332131, rs7900194, rs28371685	Losartan Efficacy
***CYP2D6***	rs16947, rs1135840, rs35742686, rs3892097, rs1065852, rs5030655, rs5030867, rs5030865(A), rs5030656, rs1065852, rs1135840, rs5030865(T), rs28371706, rs61736512, rs59421388, rs1135840, rs28371725	Metoprolol Efficacy
***CYP3A4***	rs55785340, rs35599367	Tacrolimus Dosing
***CYP3A5***	rs776746, rs10264272, rs41303343	Tacrolimus Dosing
***F7***	rs6046	Amlodipine Efficacy
***FGF5/SH2B3/EBF1***	rs1458038, rs3184504, rs4551053	Thiazide Efficacy
***GRK4***	rs2960306, rs1024323	Beta-Blocker Efficacy
***LINC00923***	rs653747	Risk of CKD
***LOC105369332***	rs2282538	Risk of CKD
***NAT2***	rs1801279, rs1801280, rs1799930, rs1799931	Hydralazine Efficacy
***NEDD4L***	rs4149601	Diuretic Efficacy
***NPHS1***	rs3814995	Angiotensin receptor blocker efficacy
***SLC3A2***	rs489381	Risk of CKD
***SLCO1B1***	rs4149056, rs4149015	Simvastatin Dosing
***SHROOM3***	rs17319721, rs4371638, rs13146355	Risk of CKD
***TPMT***	rs1800462, rs1800460 and rs1142345, rs1800460, rs1142345, rs1800584	Azathioprine Dosing
***UMOD/PDILT***	rs4293393, rs12917707, rs11864909	Risk of CKD
***VASP***	rs10995	Thiazide Efficacy
***VKORC1***	rs9923231	Warfarin Sensitivity
***YEATS4***	rs7297610	Thiazide Efficacy

CKD: Chronic kidney disease.

**Table 2:** Demographics of health care provider survey respondents and recipients.

Demographic	Respondents (%)
Total N	76 (-)
**Gender**	
Female	27 (35.5)
Male	47 (61.8)
Other or prefer not to answer	1 (1.3)
No response	1 (1.3)
**Race**	
Black or African American	1 (1.3)
American Indian or Alaska Native	1 (1.3)
Asian	23 (30.3)
Hispanic or Latino	1 (1.3)
Native Hawaiian or Pacific Islander	0 (0)
White or Caucasian	42 (55.3)
Other or prefer not to answer	5 (6.6)
No response	3 (3.9)
**Training**	
Trainee	37 (48.7)
Nephrologist	39 (51.3)
Other	0 (0)
**Health System**	
County Safety Net Hospital	9 (11.8)
University Hospital	32 (42.1)
Affiliated University Hospital with private practice model	17 (22.4)
Pediatric Hospital	3 (3.9)
Veteran Affairs Hospital	7 (9.2)
Unknown	8 (10.5)

Percentages may not total 100% due to rounding. Trainees: fellows and residents; Nephrologists: nephrologists in practice or outside an academic environment, nurse practitioner, physician assistant, or other clinician, academic attending nephrologist (majority of time spent in clinical care), academic attending nephrologist (majority of time spent in research, teaching, or administration); -, not applicable.

**Table 3:** Survey question conditions and disciplines.

Question	Condition	Discipline	Mean (SD) Agreement
1. A patient’s genetic profile can influence their risk of CKD progression	CKD	Knowledge	4.2 (0.6)
2. Genetic testing will help me to better diagnose the cause of my patient’s CKD	CKD	Action	3.6 (0.8)
3. Patients and their families can benefit from understanding genetic contributors to their CKD.	CKD	Attitude	4.0 (0.6)
4. Genetic testing of my CKD patients provides information that may change my therapeutic management of patients.	CKD	Action	3.8 (0.8)
5. Genetic testing of my CKD patients provides information that changes dialysis preparation strategies in my patients.	CKD	Action	3.3 (0.9)
6. Genetic testing for CKD provides information that will help me delay or halt the progression of CKD in my patients.	CKD	Action	3.5 (0.8)
7. The presence of 2 APOL1 risk alleles in a potential donor would impact the decision to donate a kidney for transplantion.	CKD	Action	3.8 (0.8)
8. The presence of 2 APOL1 risk alleles in a patient would impact my management of Focal Segmental Glomerulosclerosis (FSGS).	CKD	Action	3.6 (0.7)
9. I personally review and discuss a family history (taken by myself or a physician-in-training/nurse/physician extender) for every patient I meet in the hospital or in a clinic.	General	Attitude	4.1 (0.8)
10. Genetic testing is a valuable complement to a detailed family history.	General	Knowledge	3.7 (0.7)
11. Discussing genetic testing results with patients will lead to increased patient anxiety.	General	Attitude	3.1 (1.1)
12. Nephrologists should be trained to interpret and counsel patients on genetic variants that contribute to CKD.	CKD	Attitude	4.1 (0.6)
13. Genetic counselors should be trained to interpret and counsel patients on genetic variants that contribute to CKD.	CKD	Attitude	4.2 (0.6)
14. There is sufficient evidence to implement genetic testing in patients with CKD.	CKD	Knowledge	3.2 (0.9)
15. The benefits of genetic testing outweigh the risks to patients.	General	Knowledge	3.8 (0.7)
16. I feel comfortable discussing genetic test results with patients.	General	Attitude	3.1 (1.1)
17. A discussion of genetic test results is too time-consuming for a clinic encounter.	General	Attitude	3.3 (1.0)
18. Genetic testing may help me better treat my patient’s hypertension.	HTN	Knowledge	3.8 (0.8)
19. A patient’s genetic profile can influence their therapeutic response to antihypertensives.	HTN	Knowledge	4.0 (0.7)
20. Genetic testing of my HTN patients provides information that may change the antihypertensives that I prescribe.	HTN	Action	3.8 (0.9)
21. Genetic testing of my HTN patients provides information that may help me better achieve the recommended blood pressure goals.	HTN	Action	3.7 (0.8)
22. Provided no contraindications exist, I would follow the dosing suggestions of a pharmacogenomic test for a NEW prescription if the test indicated an alternate medication or dose was appropriate.	HTN	Action	4.0 (0.7)
23. Provided no contraindications exist, I would change an EXISTING prescription, one in which the patient had a stable response, in order to follow the dosing suggestions of a pharmacogenomic test if the test indicated an alternate medication or dose was appropriate.	HTN	Action	3.4 (0.9)

The averages and standard deviations were found within the context of one nonresponse for both questions 4 and 5. CKD: Chronic Kidney Disease; HTN: Hypertension.

## Supplementary Materials

The following are available online at https://www.mdpi.com/2075-1729/10/4/32/s1, Table S1: Full survey response results for all questions.
